# Prognostic Factors for Survival in Patients with High-Grade Meningioma and Recurrence-Risk Stratification for Application of Radiotherapy

**DOI:** 10.1371/journal.pone.0097108

**Published:** 2014-05-12

**Authors:** Shigeru Yamaguchi, Shunsuke Terasaka, Hiroyuki Kobayashi, Katsuyuki Asaoka, Hiroaki Motegi, Hiroshi Nishihara, Hiromi Kanno, Rikiya Onimaru, Yoichi M. Ito, Hiroki Shirato, Kiyohiro Houkin

**Affiliations:** 1 Department of Neurosurgery, Hokkaido University Graduate School of Medicine, Sapporo, Japan; 2 Department of Pathology, Hokkaido University Graduate School of Medicine, Sapporo, Japan; 3 Department of Radiation Oncology, Hokkaido University Graduate School of Medicine, Sapporo, Japan; 4 Department of Biostatistics, Hokkaido University Graduate School of Medicine, Sapporo, Japan; 5 Department of Neurosurgery, Teine-keijinkai Hospital, Sapporo, Japan; The Ohio State University Medical Center, United States of America

## Abstract

**Background:**

Radiotherapy for high-grade meningioma (HGM) is one of the essential treatment options for disease control. However, appropriate irradiation timing remains under debate. The object of this study is to discern which prognostic factors impact recurrence in HGM patients and to propose a risk-stratification system for the application of postoperative radiotherapy.

**Methods:**

We retrospectively reviewed 55 adult patients who were diagnosed with Grade II and III intracranial meningioma. Cox regression models were applied to the analysis for impact on early recurrence in HGM patients without postoperative radiotherapy.

**Results:**

Grade III malignancy (P = 0.0073) and transformed histology (P = 0.047) proved to be significantly poor prognostic factors of early recurrence by multivariate analysis. The other candidates for recurrence factors were Simpson Grade 3–5 resection, preoperative Karnofsky Performance status < = 70%, and MIB-1 labeling index > = 15%. According to these prognostic factors, postoperative HGM patients could be stratified into three recurrence-risk groups. The prognoses were significantly different between each group, as the 3-year actual recurrence-free rates were 90% in low-risk group, 31% in intermediate-risk group, and 15% in high-risk group.

**Conclusion:**

We propose recurrence-risk stratification for postoperative HGM patients using clinically available factors. Our results suggest that the prognosis for patients with high-risk HGMs is dismal, whereas HGM patients belonging to the low-risk group could have favorable prognoses. This stratification provides us with the criteria necessary to determine whether to apply adjuvant radiotherapy to postoperative HGM patients, and to also help identify potentially curable HGMs without adjuvant radiotherapy.

## Introduction

Although meningiomas have become the most common primary brain tumor and the majority of these are considered histologically benign [1], there is low incidence of high-grade meningiomas (HGMs), defined as Grade II and Grade III by WHO classification, and their biological behaviors are occasionally unpredictable [2], [3]. In particular, the aggressive nature of HGMs in the event of tumor relapse has been noted, and recurrent HGMs are generally difficult to manage.

Retrospective studies have demonstrated that adjuvant radiotherapy can contribute to a favorable prognosis for patients with HGM [2], [4]. However, the optimal timing of radiotherapy remains unclear for many clinicians. Some studies recommend that patients for whom gross total resection of the HGM cannot be achieved should receive postoperative radiotherapy [5], [6], whereas other reports recommend that all patients with HGMs should receive postoperative irradiation regardless of the extent of the resection [2], [4]. Thus, the indication of postoperative radiotherapy for HGMs is only discussed with respect to the extent of resection. However, is the extent of resection a sufficient clinical prognostic factor, especially by itself, when we make a decision regarding irradiation timing for postoperative HGM patients?

To elucidate the influence of radiotherapy on treatment outcomes and to discuss suitable irradiation timing in patients with HGMs, we rigorously reviewed the clinical factors and outcomes of HGM patients treated at our institutions and paid special consideration to radiation timing. We performed multivariate analysis of clinical and pathological factors, which are typically available in the postoperative period, leading to the identification of possible prognostic factors for the risk of recurrence for HGM patients without postoperative radiotherapy. Based on the results of this analysis, we propose a stratification of recurrence-risk. In addition, an important aim of this study was to identify the patient group that did not require postoperative radiotherapy using appropriate criteria.

## Materials and Methods

### Patients

This study was approved by the Internal Review Board on Ethical Issues of Hokkaido University Hospital and appropriate written informed consents were obtained from eligible patients. A retrospective review was performed at the Hokkaido University Hospital and our affiliated institutions on patients since 1995 that were over 20 years old with a histological diagnosis of HGM, including WHO Grade II (n = 42) and Grade III (n = 13). We referred to pathological reports to identify HGM patients, and their diagnoses were re-confirmed by senior neuropathologists (H.N. and H.K.) according to WHO 2007 criteria, as described below. Pediatric patients, spinal meningiomas, and radiation-induced meningiomas were excluded in this study.

Ultimately, there were 27 males and 28 females with a mean age of 60±15 years (range: 23–84). Regarding histological classification, Grade II meningiomas included two clear cell meningiomas and one chordoid meningioma, and Grade III meningiomas included one papillary meningioma and one rhabdoid meningioma on which we have reported previously [7]. In this study, we included patients with HGMs that were transformed from benign (Grade I) meningiomas at first presentation. Those tumors are defined as “transformed”, whereas the tumors that were diagnosed as HGM at first presentation were defined as “de novo” [8]. Ten Grade II tumors were categorized as transformed HGM; the mean interval between benign and Grade II histology was 10±9 years (range: 1–30 years). There are no cases that had progressed directly from benign to Grade III included in this series. All patients' characteristics are shown in Table 1.

**Table 1:** Descriptive statistics of study samples by postoperative radiotherapy.

	All patients (n = 55)	Early RT group (n = 19)	Deferred RT group (n = 36)	P-value^a^
Age (year), mean ± SD	60±15	58±15	62±16	0.38^b^
Gender				0.13^c^
Male	27	12	15	
Female	28	7	21	
Preoperative KPS (%)				0.59^c^
80–100%	35	13	22	
<80%	20	6	14	
Location				0.46^d^
Convexity	17	4	13	
Parasagittal/Falcial/Tentorial	20	8	12	
Sphenoid ridge	9	2	7	
Skull Base	5	3	2	
Others	4	2	2	
Tumor size (cm), mean ± SD	5.4±1.8	5.4±1.9	5.3±1.8	0.91^b^
Benign meningioma at first presentation				0.74^c^
No (de novo)	45	16	29	
Yes (transformed)	10	3	7	
Extent of Resection (Simpson Grade)				0.23^d^
Grade 1	14	4	10	
Grade 2	12	2	10	
Grade 3–5	29	13	16	
Histology				0.10^d^
Grade II	42	12	30	
Grade III	13	7	6	
MIB-1 labeling index (%), mean ± SD	11.2±7.4	12.6±7.2	10.4±7.5	0.30^b^
Median follow-up period (months)	43.9	50.1	40.3	0.62^e^
Endpoint				
Recurrence (%)	34 (62%)	11 (58%)	23 (94%)	
Death (%)	17 (31%)	9 (47%)	8 (22%)	

Abbreviations: SD, standard deviation; RT, radiotherapy.

a Comparison between early irradiation group and deferred irradiation group.

P-values were calculated by ^b^Welch t-test, ^c^Pearson's Chi-squared test, ^d^Fisher's exact test and ^e^Mann-Whiteny.

U-test.

### Clinical Parameters and Outcome Assessment

Tumor size was defined by the largest diameter of contrast enhancement on the preoperative imaging. Each patient's preoperative condition was assessed by the Karnofsky performance status (KPS). Tumor locations were categorized into five groups: convexity, found in 17 cases; parasagittal/falcine/tentorial, in 20 cases; sphenoid ridge, in 9 cases; skull base, in 5 cases; and other, in 4 cases including intraventricular (n = 2), orbital (n = 1), and interosseous (n = 1). The endpoints were recurrence-free survival (RFS) and overall survival (OS), which were measured from the time of first HGM diagnosis. In the patients with transformed HGM, their time interval from benign to high-grade was not included in the survival analysis. All patients were followed in our institutions until death or their last visit. The time of recurrence was defined as the development of either clinically and radiographically evident relapse, or tumor re-growth. Patients without event were regarded as censored observations at the last follow-up visit.

### Treatment

Simpson Grades 1 and 2 resections were designated as gross total resection confirmed by both operation record and postoperative radiographic appearance [9]. Postoperative adjuvant radiotherapy was administered to 19 patients, while the remaining 36 patients had irradiation deferred in case of relapse or tumor re-growth. The patients with postoperative radiotherapy were classified into the “early” irradiation group, and the others were classified into the “deferred” irradiation group. Postoperative radiotherapy was administered at the discretion of the physician. At the time of this analysis, 15 out of 36 patients in the deferred irradiation group had received irradiation for recurrent tumors. In terms of radiotherapy, patients were treated with X-ray based radiotherapy. The range of cumulative irradiation dose were from 50 Gy to 60 Gy using 2.0 Gy as the daily dose. Patients with HGM who were treated by other radiotherapies, such as gamma-knife or Boron Neutron Capture therapy (BNCT), are not included in this series.

### Pathological Examination

All patients were re-evaluated to confirm the pathological diagnosis according to WHO 2007 criteria by senior neuropathologists. They counted mitoses per 10 high-power fields (HPFs, ×400) and the 5 prognostic histological parameters of hypercellularity, macronucleoli, small cell formation, patternless architecture and necrosis as 0 (no) or 1 (yes). The sum of each parameter was designated as an atypical score. Cases with 4 or more mitoses per 10 HPFs or with an atypical score greater or equal to 3 correspond to atypical meningioma. Cases with an obviously malignant cytology resembling that of carcinoma, melanoma, high-grade sarcoma, or a markedly elevated mitotic index (20 or more mitoses per 10 HPFs) correspond to anaplastic meningioma [10]. Cellular proliferation was assessed using the MIB-1 labeling index by immunohistochemistry. The quantification of the MIB-1 labeling index was performed by H.K., who was blinded to the clinical information. Eventually, MIB-1 labeling was made available to index of 50 out of 55 cases.

### Statistical Analysis

All statistical analyses were carried out in R statistical environment version 3.0.2. Continuous variable data were expressed with standard deviation (SD). The mean of continuous variables was compared by Welch two sample t-tests, the median of continuous variables was compared by Mann-Whitney U test or Kruskal-Wallis test, and the distribution of categorical variables was compared by Pearson's Chi-squared test or Fisher's exact test according to the counts of expected frequencies. Estimated survival curves were shown by Kaplan-Meier method, and a log-rank test was used for the comparison.

**Figure 1:**
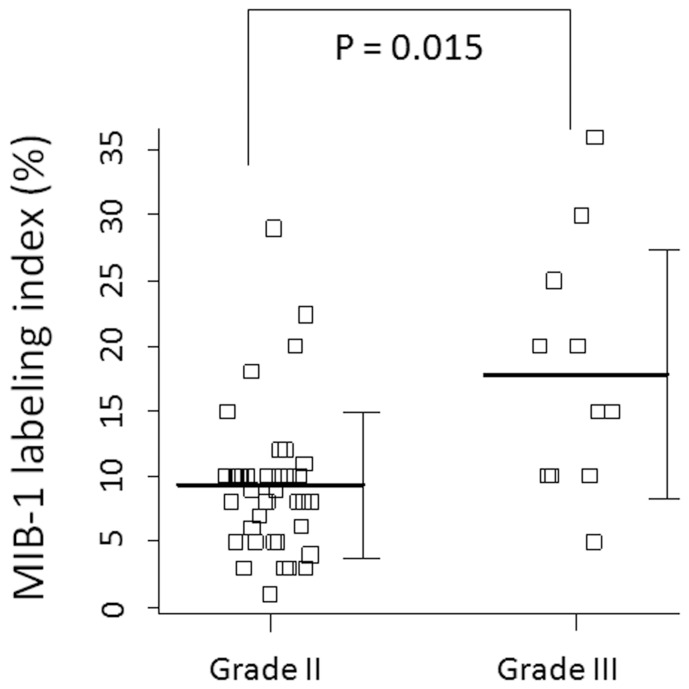
The MIB-1 labeling index of Grade II and Grade III meningioma. The mean MIB-1 labeling index of Grade II and Grade III meningioma are 9.3% and 17.8%, respectively, and these mean value are significantly different (p = 0.015). The bars represent the mean values and standard deviations.

To analyze prognostic factors for the risk of recurrence in the deferred irradiation group, the patient and the treatment characteristics were evaluated for association with the time to recurrence using Cox proportional hazards regression model. The analyzed characteristics included the patient's age, gender, preoperative KPS, previous diagnosis of meningioma, location of the tumor, extent of resection, MIB-1 labeling index, and the histological grade. A hazard ratio, with 95% confidence intervals (CIs) from a Cox model, summarized the effect; a non-parametric CI was calculated by the Greenwood formula. In multivariate analysis, the factors for which the *P*-value was below 0.1 in univariate analysis were selected. The factor of the MIB-1 labeling index could not be applied in multivariate analysis due to significant correlation with the histological grade (P = 0.015, Fig. 1). Statistical significance was given to p-values <0.05.

## Results

### Patient characteristics

Table 1 shows the patient characteristics between the early irradiation group and the deferred irradiation group. In comparison to the deferred irradiation group, the number of Grade III meningioma patients is higher in the early irradiation group, but the difference is not statistically significant (P = 0.10). There were no significant differences with respect to other clinical factors, nor to the extent of the resection. 34 out of the 55 tumors were found to have recurred and 17 patients died as a result of tumor progression. The median follow-up period of all patients was 43.9 months (range: 3.1–182.9 months), and there was no significant difference in median follow-up period between the two groups (P = 0.62). 21 out of 36 patients in the deferred irradiation group did not require irradiation at the time of this analysis. The median follow-up period of these 21 patients was 36.4 months.

### Prognostic factors in deferred irradiation group

To identify which clinical factors influenced the recurrence of HGMs, we analyzed the deferred irradiation group using the Cox model (n = 36, Table 2). According to multivariate analysis, two parameters were found to be significant poor prognostic factors of early recurrence: Grade III malignancy (P = 0.0073) and transformed histology (P = 0.047). Although Simpson Grade 3–5 resection was one of the candidates of poor prognostic factors in univariate analysis (P = 0.0034), the extent of resection was not found to influence tumor recurrence in multivariate analysis (P = 0.82). The other possible poor prognostic factor was poor preoperative KPS (P = 0.019, in univariate analysis). Although we could not apply the MIB-1 labeling index of the tumor in multivariate analysis, univariate analysis indicated that a high MIB-1 labeling index, defined as more than 15%, might be a possible candidate for a prognostic factor for early recurrence (P = 0.020).

**Table 2:** Cox regression Hazard model on RECURRENCE FREE SURVIVAL in deferred irradiation group.

		Univariate analysis			Multivariate analysis		
Factors		Hazard ratio	95% CI	P-value	Factors	Hazard ratio	95% CI
Age*		1.017	0.988–1.05	0.26			
Gende	Male	0.784	0.333–1.84	0.58			
Location	Not convexity	1.852	0.679–5.05	0.229			
Preoperative KPS (%)	80–100%	0.360	0.153–0.844	0.019	0.421	0.166–1.07	0.069
Tumor size	>6.0 cm	1.551	0.632–3.81	0.338			
Histology	Grade III	4.648	1.74–12.4	0.0022	6.68	1.67–26.8	0.0073
Histology at presentation	transformed	3.16	1.26–7.95	0.0144	4.33	1.02–18.4	0.047
Extent of resection (Simpson grade)	Grade 3–5	3.95	1.58–9.89	0.0034	1.167	0.317–4.30	0.82
MIB-1 labeling index (%) (n = 35)	10–15%	0.975	0.323–2.95	0.96	NA	NA	NA
	>15%	3.683	1.23–11.0	0.020	NA	NA	NA

Abbreviations: CI, confidence interval; KPS, Karnofsky performance status; NA, no assessment.

*continuous variable.

### Recurrence-risk stratification

Based on the analyzed results of the Cox model, we propose to stratify the recurrence-risk group according to these prognostic factors (Table 3). For the high-risk group, two classifiers are selected that were identified as significant poor prognostic factors by multivariate analysis: Grade III malignancy, and transformed histology. For the intermediate-risk group, three prognostic factors are selected as classifiers based on univariate analysis as follows: the patients with poor preoperative KPS, tumors with Simpson grade 3–5 resection, and high proliferative tumors suggested by high MIB-1 labeling index (more than 15%). The tumors that meet any of the above criteria are stratified into each recurrence-risk group, and the patients whose clinical and pathological characteristics do not match the above criteria are stratified into a low-risk group.

**Table 3:** Recurrence-risk stratification of high-grade meningioma.

Risk group	Classifiers
**High-risk group**	1 Grade III malignancy
	2 Transformed histology
**Intermediate-risk group**	1 Poor preoperative KPS score (less than 70%)
	2 Simpson grade 3–5 resection
	3 High MIB-1 labeling index (more than 15%)
**Low-risk group**	None of matched above factors

Abbreviation: KPS, Karnofsky performance status.

**Figure 2:**
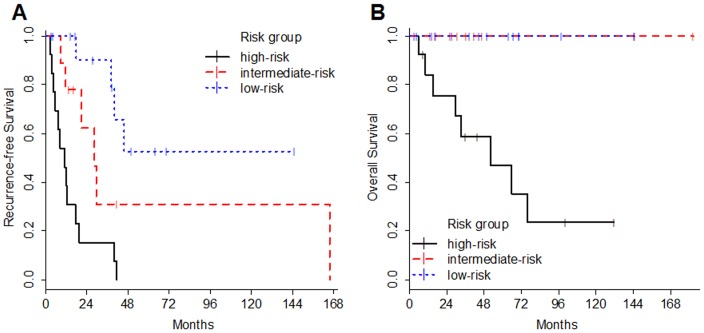
Kaplan-Meier estimates based on the recurrence-risk stratification. The graphs show recurrence-free survival (A) and overall survival (B) according to the recurrence-risk stratification defined as prognostic factors. Prognosis shows a significant difference in both RFS and OS among the recurrence-risk stratified groups (p<0.001 in PFS, P = 0.001 in OS).

Figure 2 shows Kaplan-Meier curves of the patients in the deferred irradiation group according to the recurrence-risk stratification we propose. The prognosis shows a significant difference not only in RFS but also in OS among the recurrence-risk stratified groups (p<0.001 in PFS, P = 0.001 in OS). The 3-year actual recurrence-free rates of the low-risk, intermediate-risk, and high-risk groups were 90%, 31%, and 15%, respectively. In the intermediate-risk group, the median RFS is 28.4 months. Although the RFS of the intermediate-risk group was poor compared to the low-risk group, all patients who were stratified in intermediate-risk and low-risk group have been alive through follow-up periods. Finally, the prognosis of the high-risk group was dismal. The median RFS and OS of the high-risk group are 11.2 months and 52.1 months, respectively.

**Figure 3:**
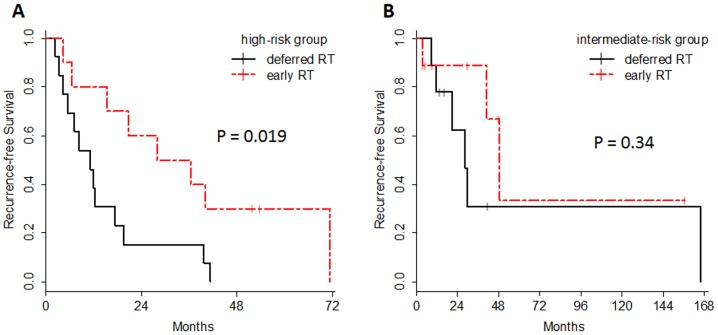
Recurrence-free survival analysis according to postoperative irradiation. Kaplan-Meier estimates of recurrence-free survival are illustrated according to the treatment option of postoperative irradiation in high-risk group (A) and intermediate-risk group (B). In high-risk group, the prognosis of the patients with early irradiation was significantly better (P = 0.019), whereas there were no significant prognostic differences between early irradiation and deferred irradiation in the intermediate-risk HGMs (P = 0.34). RT stands for radiation therapy.

In addition, when the patients who received early irradiation had been assigned to this recurrence-risk stratification, 10 out of 19 tumors fell into the high-risk group, and 9 out of 19 tumors were in the intermediate-risk group. Figure 3 shows the RFS in the high-risk group and intermediate-risk group according to the postoperative radiation. As clearly shown, the prognosis of the patients with high-risk HGMs who were treated by early irradiation was significantly better (P = 0.019), whereas there were no significant prognostic differences between early irradiation and deferred irradiation in the intermediate-risk HGMs (P = 0.34).

## Discussion

Since radical resection of meningioma is widely agreed to cause an improvement of prognosis [11], neurosurgeons always attempt to resect the tumor at the highest possible extent irrespective of histological subtype or tumor location. Although some promising antineoplastic agents, such as trabectedin [12] or histone deacetylase inhibitors [13], are being used in preclinical studies, commonly acceptable chemotherapies for HGMs are currently unavailable. Therefore, radiotherapy remains the sole treatment option after surgical resection of HGMs, and the timing of radiotherapy is of great concern to physicians and has been discussed in several retrospective analyses [2], [4], [5], [6].

There is no doubt that radiotherapy for HGMs is beneficial for controlling tumor recurrence and has demonstrated improvement in patient prognosis [2], [4]. However, compared to other malignant brain tumors such as high-grade gliomas and medulloblastomas, the role of radiotherapy for HGMs remains ambiguous. Previously, some studies suggested that patients with atypical meningiomas for whom gross total resection is possible do not necessarily need postoperative radiotherapy [5], [6]. In addition, Pearson et al. pointed out that the incidence of atypical meningiomas increased dramatically after 2004 due to the reclassification of WHO criteria [6]. Our series also show this trend, as 39 (71%) out of the 55 cases were diagnosed as HGMs after 2004. This fact might indicate that recent cases diagnosed as HGM might include cases that did not meet the old criteria, suggesting in turn that the number of surgically curable HGMs may have recently increased.

The main aim of this study is to attempt to identify the prognostic risk factors of early recurrence that are available at the time physicians decide whether postoperative irradiation should be performed. To eliminate the influence of radiotherapy, we specifically focused on HGM patients who did not received postoperative radiotherapy at primary HGM diagnosis. Thereafter, we stratified our patient pool into three recurrence-risk groups according to these factors, which were identified by multivariate and univariate analyses, and we validated the survival effect for each of these groups. Although this novel approach is debatable, we propose that it can provide some clues for the treatment strategy of this rare disease.

Through this analysis, we were able to identify two significant risk factors: Grade III malignancy and transformed histology. In terms of Grade III meningioma, previous reports evidently recommended postoperative radiotherapy regardless of the extent of resection [14], [15]. Durand et al. evaluated the prognostic factors for high-grade meningioma on 199 adult patients. Although no significant difference was found in overall survival rate between the patients who had and had not received radiation adjuvant treatment, it was found that only the prognosis of Grade III meningioma could be improved by postoperative radiotherapy [16]. These results are consistent with our analysis.

The other significant poor risk factor is transformed malignancy. In our series, all transformed HGM cases ranged from benign to atypical. Nevertheless, the prognosis of these patients was significantly poor, as was that of the Grade III meningioma patients. With respect to glioblastoma, secondary malignancy is representative of a good prognostic factor [17], whereas the malignant transformation exhibited contrasting findings for HGM. This poor prognostic factor was also recognized by two previous studies [8], [18]. Interestingly, Krayenbühl et al. demonstrated the significant differences of histological characters, in addition to cytogenetic findings between “de novo” subgroup and “transformed” subgroup. They hypothesized that the “transformed” HGMs could comprise distinct subgroups of aggressive meningiomas compared to “de novo” HGMs [8]. In addition, Yang et al. reported that tumors with malignant transformation had a higher percentage of p53 overexpression than “de novo” tumors [18]. Their findings are consistent with our results, and can provide the biological clues toward a better understanding of the poor prognosis of this subpopulation.

For the classifiers of the intermediate-risk group, three risk factors were designated based on univariate analyses: patients' poor preoperative KPS, incomplete tumor resection, and tumors with high MIB-1 labeling indices. Our series failed to demonstrate a significant beneficial effect from gross total resection in multivariate analysis, suggesting that the extent of resection is not always a definitive prognostic factor for HGM patients. In addition, we adopted the MIB-1 labeling index as a prognostic factor by histological aspect. It is well known that the MIB-1 labeling index is routinely performed worldwide and recognized as one of the most reliable markers of proliferative tumor activity [19]. Compared to Grade III meningioma, it is commonly recognized that the diagnosis criteria of Grade II meningiomas are highly controversial despite the objective criteria of WHO classification. In actuality, the difference of mean MIB-1 labeling index among the studies was significant, ranging from 3.2%[18] to 15.81% [20]. To complement this interinstitutional or interobserver difference, the “high MIB-1 labeling index” became a proper objective factor to identify the tumors that might pose a potential risk for early recurrence.

For the treatment of high-risk HGM patients, we advocate postoperative radiotherapy regardless of the extent of resection. As shown in Figure 3A, early irradiation could contribute to prolonged recurrence-free survival of the patients with high-risk HGM. On the other hand, patients with low-risk HGMs should not be given up-front radiotherapy. Low-risk HGMs might be curable without irradiation and the patients may ultimately remain free of recurrent disease, as with the patients who undergo complete resection of benign meningiomas. In addition, in the instance that low-risk HGMs relapse, our data suggests that the recurrent tumor could be regulated via salvage operation or radiotherapy. Compared to the high-risk and low-risk groups, the biological behaviors and clinical courses of the intermediate-risk HGMs are heterogeneous. It is ambiguous whether the patients in the intermediate-risk group should receive postoperative radiotherapy. The Kaplan-Meier curves in Fig. 3B provide a visual representation of the recurrence pattern. These curves indicate that the intermediate-risk group included tumors with a high possibility of early recurrence, especially for the first three years following diagnosis, as well as tumors that are potentially curable without irradiation.

## Conclusions

Although the influence of irradiation will likely be difficult to fully elucidate in a single-institution series, our scrupulous analysis provides a clue as to how to manage treatment for HGM patients. We propose recurrence-risk stratification using available clinical and histopathological factors for the purpose of making decisions regarding radiotherapy for postoperative HGM patients. Multicenter reviews and prospective studies are necessary to evaluate this stratification system for validity.
